# Qualitative Evaluation of Binding States of Lipid Membranes to Mesoporous Silica Microspheres via Single-Particle Inductively Coupled Plasma Mass Spectrometry

**DOI:** 10.3390/molecules30173621

**Published:** 2025-09-04

**Authors:** Shin-ichi Miyashita, Toshihiko Ogura, Shun-ichi Matsuura, Toshiyuki Takagi, Eriko Fukuda

**Affiliations:** 1 National Metrology Institute of Japan (NMIJ), National Institute of Advanced Industrial Science and Technology (AIST), 1-1-1 Umezono, Tsukuba 305-8563, Ibaraki, Japan; shinichi-miyashita@aist.go.jp; 2 Health and Medical Research Institute, National Institute of Advanced Industrial Science and Technology (AIST), 1-1-1 Higashi, Tsukuba 305-8566, Ibaraki, Japan; t-ogura@aist.go.jp; 3 Research Institute for Chemical Process Technology, National Institute of Advanced Industrial Science and Technology (AIST), 4-2-1 Nigatake, Miyagino-ku, Sendai 983-8551, Miyagi, Japan; matsuura-shunichi@aist.go.jp; 4 Cellular and Molecular Biotechnology Research Institute, National Institute of Advanced Industrial Science and Technology (AIST), 1-1-1 Higashi, Tsukuba 305-8565, Ibaraki, Japan; t.takagi@aist.go.jp

**Keywords:** lipid membrane, liposome, nanodisc, mesoporous silica microsphere, single-particle ICP-MS, confocal laser Raman microscopy

## Abstract

Single-particle inductively coupled plasma mass spectrometry (spICP-MS) offers the unprecedented advantage of sensitive and selective detection of individual particles based on their constituent elements. It has been applied to the qualitative/quantitative evaluation of nonporous/mesoporous particles ranging from the nanoscale to the microscale and, recently, targeted proteins bound to particles. However, lipid membranes bound to particles have not been explored as potential targets for spICP-MS, despite its analytical potential. To address this, we investigated the applicability of spICP-MS for evaluating the binding states of two different types of lipid membranes (liposomes, i.e., phospholipid bilayer-based spherical vesicles, and nanodiscs comprising a disc-shaped phospholipid bilayer and membrane scaffold protein) to mesoporous silica microspheres (SBA24). The presence of bound liposomes and nanodiscs was confirmed using spICP-MS, which selectively monitored the derived P as a marker element. The presence of bound liposomes was confirmed by confocal laser Raman microscopy. Our findings demonstrate that spICP-MS can be used to qualitatively evaluate the binding states of lipid membranes to mesoporous SiO_2_ microspheres. This method offers a new platform for evaluating the effectiveness of particles as carriers of biomolecules (lipid membranes) and provides valuable insights into biomedical research and quality control in related industries.

## 1. Introduction

Lipid membranes, which are fundamental structures of all cells, are primarily composed of lipids and proteins that form selectively permeable barriers [1,2]. Liposomes and nanodiscs are artificial systems that mimic biological membranes and offer unique tools for research and drug delivery [3,4,5,6]. Liposomes are composed of phospholipid molecules that spontaneously self-assemble into spherically closed bilayer membranes [4], in which the polar heads are hydrated with water and the hydrophobic hydrocarbon chains of each leaflet of the bilayer core interact with each other [7,8]. The thickness of the lipid bilayer of the liposomes is approximately 5 nm [4]. Typical phospholipids used to prepare liposomes include phosphatidylcholines, phosphatidylethanolamines (PEs), and phosphatidylglycerols (PGs) [4]. The phospholipid composition of liposomes defines the membrane surface properties; for example, the interaction with charged supports, interaction with membrane proteins, permeability, and wetting depend significantly on the presence of charged lipids [9,10]. Nanodiscs are organized as a disc-shaped phospholipid bilayer whose perimeter is circumscribed by a membrane scaffold protein (MSP), which is typically a member of the exchangeable apolipoprotein family [11,12]. Numerous hydrophobic bioactive agents have been efficiently solubilized in nanodiscs via integration into the hydrophobic milieu of a particle’s lipid bilayer, yielding a largely homogenous population of particles of approximately 10 nm in diameter [11,12].

The binding/adsorption of biomolecules such as proteins [13] and lipid membranes [4] on porous microspheres has been extensively investigated. For example, porous silica (SiO_2_) microspheres possess an ordered pore network with a large surface area and pore volume; in particular, the surface area was calculated to be approximately four orders of magnitude larger than that of solid microspheres of equivalent size [14,15]. Therefore, these microspheres have been used as biomolecular carriers [16,17]. Analytical techniques play a significant role in studying the properties of porous microspheres and in understanding the interactions between porous microspheres and biomolecules.

Characterization studies of mesoporous microspheres typically involve particle-size measurement, overall porosity determination, and evaluation of their effectiveness as carriers of biomolecules such as proteins [14]. For this purpose, various particle measurement techniques, such as laser diffraction, scattering, and dynamic light scattering, have been employed [18]. Single-particle inductively coupled plasma mass spectrometry (spICP-MS)—an element-specific particle sizing/counting technique—has the unprecedented advantage of the sensitive and selective detection of individual particles based on their constituent elements [19]. It has been applied to the qualitative/quantitative evaluation of nonporous/mesoporous particles ranging from nano- to micrometer sizes [20] and recently targeted proteins bound to particles [21]. Despite its analytical potential, lipid membranes bound to particles have not been explored as potential targets for spICP-MS.

**Figure 1:**
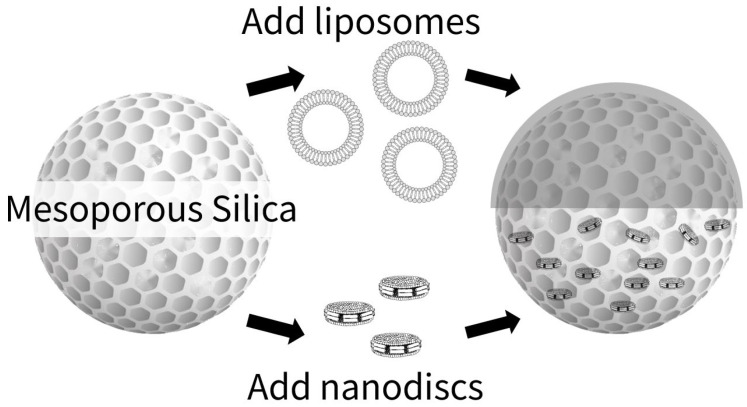
Schematic representation of liposomes and nanodiscs binding to mesoporous silica microspheres (SBA24). The upper part shows liposomes rupturing and spreading to cover the SBA24 surface, whereas the lower part shows nanodiscs binding to the SBA24 surface and/or penetrating its mesopores. Liposomes are spherical phospholipid bilayer vesicles, whereas nanodiscs are disc-shaped phospholipid bilayers stabilized by two membrane scaffold protein (MSP) belts. Note that this is a conceptual illustration (not to scale).

In this study, we investigated the applicability of spICP-MS for evaluating the binding states of lipid membranes to mesoporous SiO_2_ microspheres (Figure 1). For this purpose, we used two different types of lipid membranes (liposomes and nanodiscs) and selectively monitored the P derived from the lipid membranes bound to mesoporous SiO_2_ microspheres as a marker element in spICP-MS. To validate the spICP-MS results, confocal laser Raman microscopy (CLRM) was used to confirm the presence of lipid membranes bound to the mesoporous SiO_2_ microspheres.

## 2. Results and Discussion

### 2.1. Qualitative Evaluation of Liposomes Bound to Mesoporous SiO_2_ Microspheres via spICP-MS

**Figure 2:**
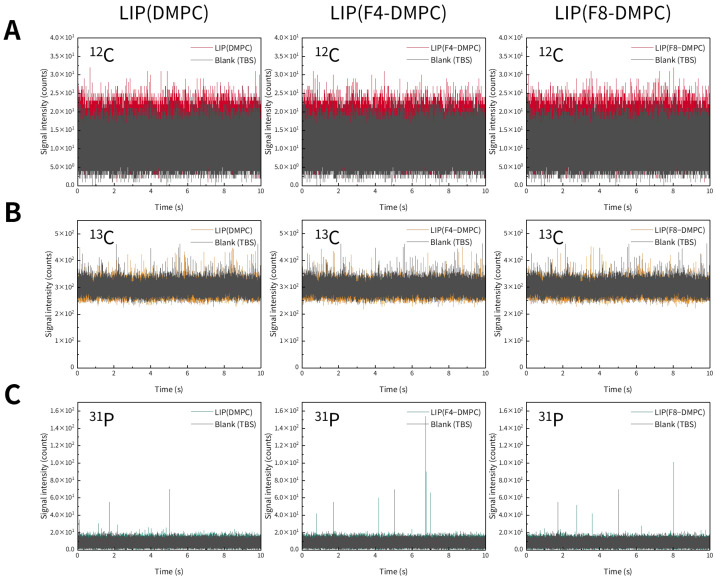
Representative time-resolved profiles for three types of liposomes [LIP(DMPC), LIP(F4-DMPC), and LIP(F8-DMPC)] suspended in TBS, obtained via spICP-MS while monitoring ^12^C (**A**), ^13^C (**B**), and ^31^P (**C**) individually.

**Figure 3:**
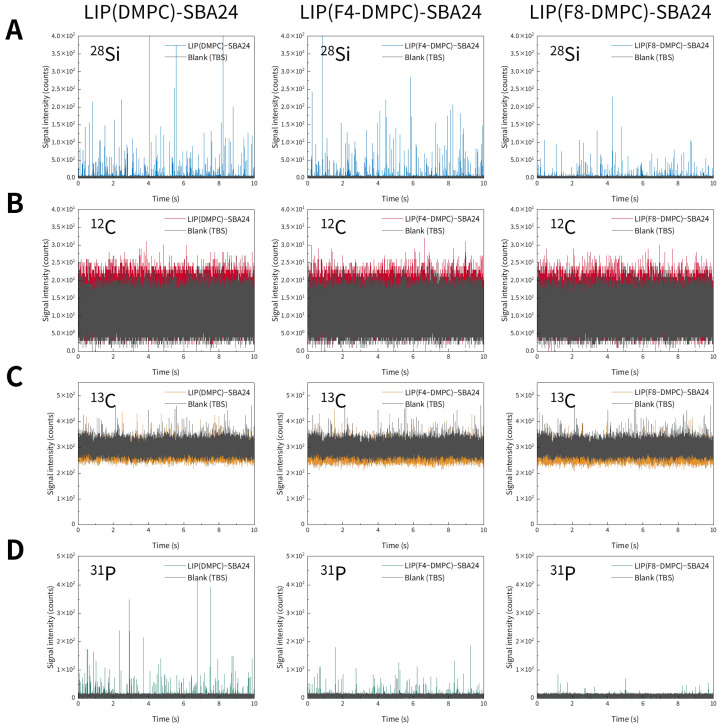
Representative time-resolved profiles for mesoporous SiO_2_ microspheres (SBA24) with the bound liposomes [LIP(DMPC), LIP(F4-DMPC), and LIP(F8-DMPC)] suspended in TBS, obtained via spICP-MS while monitoring ^28^Si (**A**), ^12^C (**B**), ^13^C (**C**), and ^31^P (**D**) individually.

We first investigated whether liposomes could be detected using spICP-MS. Representative time-resolved profiles of three types of liposomes obtained via spICP-MS are shown in Figure 2. We monitored ^12^C, ^13^C, and ^31^P using ICP-MS, targeting the C and P contained in the liposomes. However, no distinct transient signals originating from liposomes were observed for any of the monitored elements (Figure 2). Next, we examined whether the liposomes immobilized on mesoporous SiO_2_ microspheres (SBA24) could be detected by spICP-MS. Representative time-resolved profiles of SBA24 with bound liposomes obtained via spICP-MS are shown in Figure 3. We monitored ^28^Si from SBA24 and ^12^C, ^13^C, and ^31^P from the liposomes. Clear transient signals were obtained for ^28^Si originating from SBA24 and ^31^P originating from the liposomes (Figure 3A,D). However, similar to the results for the liposomes alone, no distinct transient signals derived from the liposomes were observed for ^12^C and ^13^C (Figure 3B,C). Notably, the signal intensities of ^13^C, which is less abundant, were higher than those of ^12^C, the most abundant isotope. This is because the cell gas (He) flow rate for ^12^C (5.0 mL/min) was significantly higher than that for ^13^C (0.0 mL/min; see Table 1). This difference in flow rate was employed because of the high ionization potential of C and the presence of C in both the Ar used to generate the plasma (primarily as CO_2_ impurities) and in reagents, including acids and water. These results indicate that spICP-MS with selective monitoring of the P contained in liposomes enables evaluation of the binding states of liposomes to porous particles.

Interestingly, the frequency of the transient signals for ^31^P originating from the liposomes varied significantly depending on the liposome type. Specifically, liposomes composed of 1,2-dimyristoyl-sn-glycero-3-phosphocholine (LIP(DMPC)) exhibited the highest signal frequency, followed by those composed of partially fluorinated DMPC (LIP(F4-DMPC)), and those composed of highly fluorinated DMPC (LIP(F8-DMPC)) exhibited the lowest frequency. This trend was confirmed by the CLRM results, as described later (Section 2.3). As shown in Table 2, LIP(F8-DMPC) had a diameter more than twice those of the other two types of liposomes, which may have been a contributing factor. These results suggest that the binding states of liposomes to SBA24 depend on their properties (e.g., diameter).

In spICP-MS, the peaks are the transient signals generated by individual particles as they are detected by the MS, and their signal-to-noise (S/N) ratios are crucial for distinguishing these particle signals from the constant background signal and other interferences. A high S/N, achieved by a low background and a strong particle signal, enables clear detection of particles and accurate determination of particle masses. In this study, three consecutive 60 s measurements were performed on SBA24 with the lipid membranes, and the S/N ratios were calculated from the maximum peak intensities of the peaks exceeding the mean blank (Tris-buffered saline, TBS only) signal intensity plus three standard deviations (3*σ*) and the mean blank signal intensity. Hence, the ^31^P S/N ratios (means ± standard deviations) of LIP(DMPC)-SBA24, LIP(F4-DMPC)-SBA24, and LIP(F8-DMPC)-SBA24 were calculated to be 8.7 ± 0.2 (*n* = 752–897), 7.6 ± 0.2 (*n* = 447–481), and 6.6 ± 0.5 (*n* = 61–80), respectively, from all the peaks obtained for each. As these S/N ratios were comparable and sufficiently high—even for LIP(F8-DMPC), which had a lower peak frequency (Figure 3D)—clear detection of the liposomes bound to SBA24 and accurate determination of their elemental (P) masses per particle were achieved, as described below.

**Figure 4:**
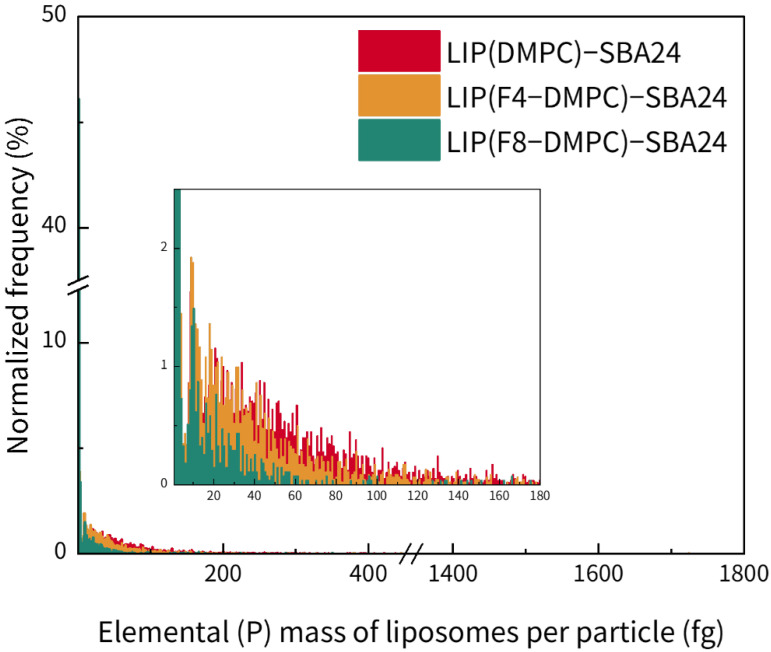
Elemental (P) masses of liposomes [LIP(DMPC), LIP(F4-DMPC), and LIP(F8-DMPC)] bound to each mesoporous SiO_2_ microsphere (SBA24). The measurements were performed using spICP-MS with ^31^P monitoring based on a conventional calibration approach using a phosphate ion standard solution. Inset: Enlarged view.

To facilitate comparisons between the results for the different types of liposomes bound to SBA24, the elemental (P) masses of the bound liposomes per particle were calculated, as shown in Figure 4. In detail, the elemental masses (means ± standard deviations, *n* = 3) of LIP(DMPC), LIP(F4-DMPC), and LIP(F8-DMPC) per SBA24 particle were calculated as (34.5 ± 1.8), (21.5 ± 1.7), and (5.9 ± 0.8) fg, respectively. The decrease in the binding amount per particle followed the same trend as that observed in the time-resolved profiles for SBA24 with the bound liposomes (Figure 3D), with LIP(DMPC), LIP(F4-DMPC), and LIP(F8-DMPC) exhibiting the highest, intermediate, and lowest frequencies, respectively.

### 2.2. Qualitative Evaluation of Nanodiscs Bound to Mesoporous SiO_2_ Microspheres via spICP-MS

**Figure 5:**
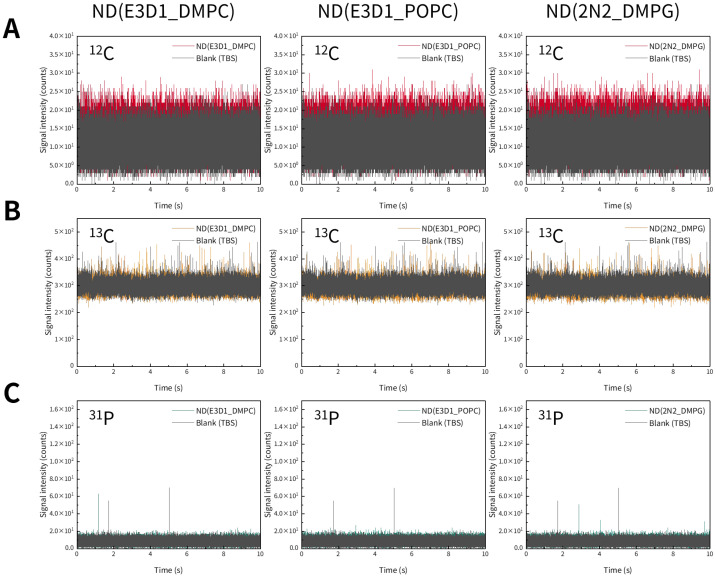
Representative time-resolved profiles for three types of nanodiscs [ND(E3D1_DMPC), ND(E3D1_POPC), and ND(2N2_DMPG)] suspended in TBS, obtained via spICP-MS while monitoring ^12^C (**A**), ^13^C (**B**), and ^31^P (**C**) individually.

**Figure 6:**
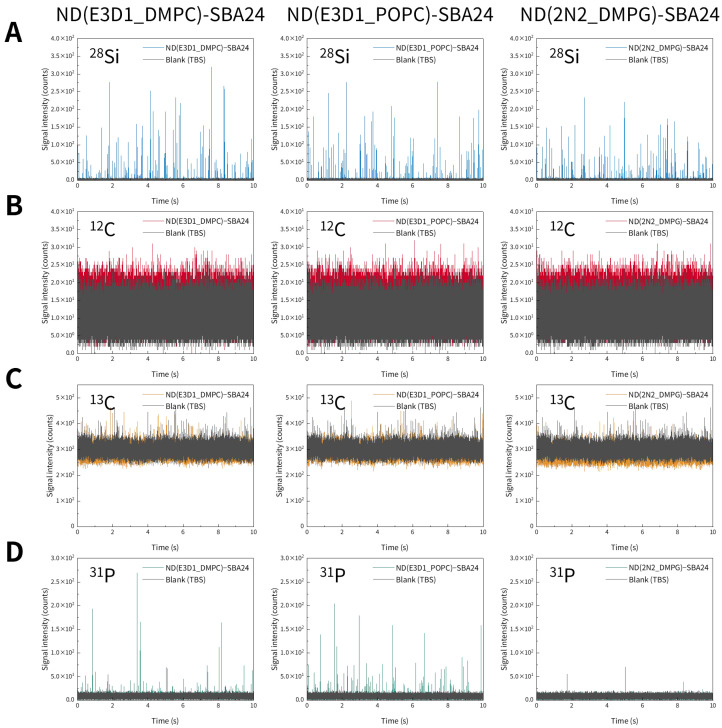
Representative time-resolved profiles for mesoporous SiO_2_ microspheres (SBA24) with the bound nanodiscs [ND(E3D1_DMPC), ND(E3D1_POPC), and ND(2N2_DMPG)] suspended in TBS, obtained via spICP-MS while monitoring ^28^Si (**A**), ^12^C (**B**), ^13^C (**C**), and ^31^P (**D**) individually.

As with the liposomes described above, we investigated whether nanodiscs could be detected by spICP-MS. Representative time-resolved profiles of three types of nanodiscs obtained via spICP-MS are shown in Figure 5. We monitored ^12^C, ^13^C, and ^31^P using ICP-MS, targeting the C and P contained in the nanodiscs. However, no distinct transient signals originating from the nanodiscs were observed for any of the monitored elements (Figure 5). Next, we examined whether nanodiscs immobilized on mesoporous SiO_2_ microspheres (SBA24) could be detected by spICP-MS. Representative time-resolved profiles of SBA24 with bound nanodiscs obtained via spICP-MS are shown in Figure 6. We monitored ^28^Si in SBA24 and ^12^C, ^13^C, and ^31^P in the nanodiscs. Clear transient signals were obtained for ^28^Si originating from SBA24 and ^31^P originating from the nanodiscs (Figure 6A,D). However, similar to the results obtained for the nanodiscs alone, no distinct transient signals derived from the nanodiscs were observed for ^12^C and ^13^C (Figure 6B,C). The reason why the signal intensities of ^13^C, which is less abundant, were higher than those of ^12^C, the most abundant isotope, is the same as in the case of the liposomes (mentioned above). These results indicate that spICP-MS with selective monitoring of the P contained in nanodiscs enables evaluation of the binding states of nanodiscs to porous particles.

As was the case with the liposomes, the frequency of the ^31^P transient signals originating from the nanodiscs varied significantly depending on the type of nanodisc used. Specifically, ND(E3D1_DMPC) and ND(E3D1_POPC) exhibited almost comparable signal frequencies, whereas ND(2N2_DMPG) exhibited almost no detectable signal. As shown in Table 3, ND(2N2_DMPG) had a larger diameter than the other two types of nanodiscs, which may have been a contributing factor. Moreover, the MSP of ND(2N2_DMPG) was MSP2N2-His, whereas that of the other two nanodiscs was MSP1E3D1-His, which also could have contributed to the difference. These results suggest that the binding states of the nanodiscs to SBA24 depend on their properties (e.g., diameter and MSP).

The ^31^P S/N ratios (means ± standard deviations) were calculated in the same manner as for the liposomes bound to SBA24, and those of ND(E3D1_DMPC)-SBA24, ND(E3D1_POPC)-SBA24, and ND(2N2_DMPG)-SBA24 were found to be 6.4 ± 0.2 (*n* = 200–244), 7.5 ± 0.3 (*n* = 367–440), and 6.1 ± 2.6 (*n* = 1–5), respectively. Because these S/N ratios were comparable and sufficiently high—even for ND(2N2_DMPG), which had lower peak frequency (Figure 6D)—clear detection of the nanodiscs bound to SBA24 and accurate determination of their elemental (P) masses were achieved, as described below.

**Figure 7:**
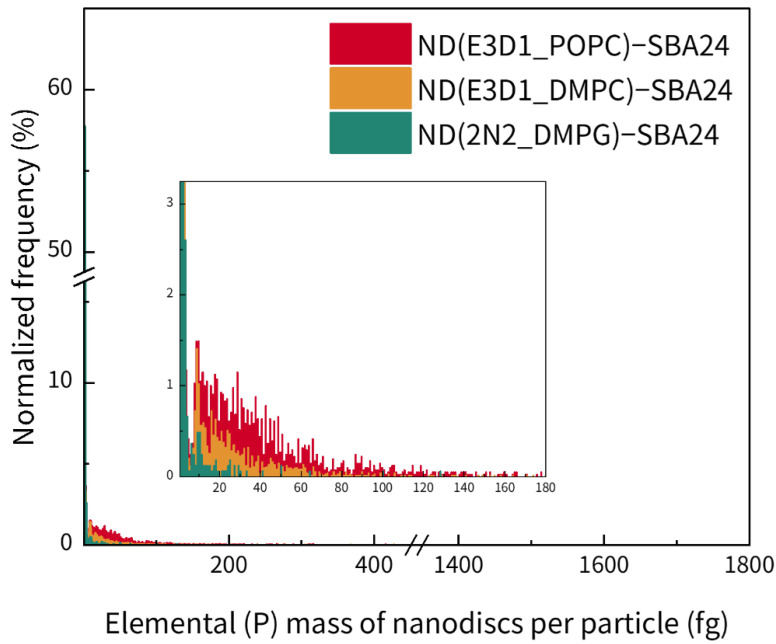
Elemental (P) masses of nanodiscs [ND(E3D1_POPC), ND(E3D1_DMPC), and ND(2N2_DMPG)] bound to each mesoporous SiO_2_ microsphere (SBA24). The measurements were performed using spICP-MS with ^31^P monitoring based on a conventional calibration approach using a phosphate ion standard solution. Inset: Enlarged view.

In the same manner as for the liposomes bound to SBA24, the elemental (P) masses of the bound nanodiscs per particle were calculated (Figure 7). Consequently, the elemental masses of ND(E3D1_DMPC), ND(E3D1_POPC), and ND(2N2_DMPG) per SBA24 particle were calculated to be (6.2 ± 0.6), (20.4 ± 1.5), and (1.6 ± 0.2) fg, respectively. Although comparison of the time-resolved profiles for SBA24 with the bound nanodiscs (Figure 6D) suggested that ND(E3D1_DMPC) and ND(E3D1_POPC) exhibited almost similar signal frequencies, with ND(2N2_DMPG) showing almost no detectable signals, further analysis of the elemental (P) masses of the nanodiscs per particle calculated as a quantitative metric (Figure 7), together with a comparison based on their mean values, demonstrated that ND(E3D1_POPC) possessed a significantly higher binding amount per particle than ND(E3D1_DMPC), with ND(E3D1_POPC) having the lowest binding amount.

### 2.3. Qualitative Evaluation of Liposomes Bound to Mesoporous SiO_2_ Microspheres via CLRM

**Figure 8:**
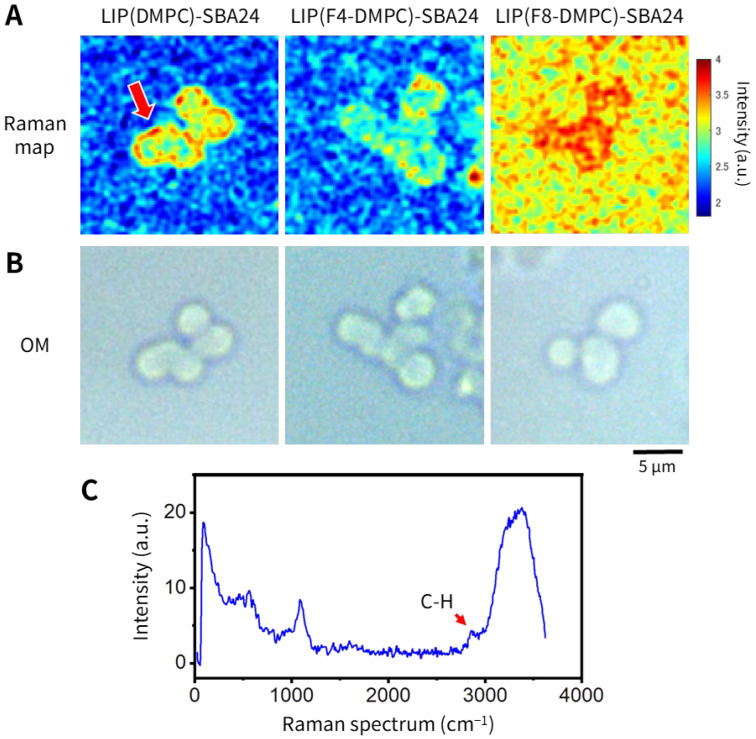
Raman spectrum map (**A**), OM images (**B**), and Raman spectrum (**C**) of mesoporous SiO_2_ microspheres (SBA24) with the bound liposomes [LIP(DMPC), LIP(F4-DMPC), and LIP(F8-DMPC)]. The red arrow in (**A**) indicates the area of strong C–H peaks surrounding the SBA24 particles, and the red arrow in (**C**) indicates a peak resulting from C–H bonds in liposome-derived lipids.

The presence of lipid membranes bound to mesoporous SiO_2_ microspheres (SBA24) was confirmed using CLRM with liposomes as representative lipid membranes. CLRM allows compositional analysis of samples by measuring the Raman spectrum obtained when a sample is irradiated with laser light [21,22,23]. In particular, during the analysis of biological samples, portions of lipids and proteins in the samples can be identified from peaks in the Raman spectrum [24,25,26]. In this study, we analyzed the Raman spectra of three different types of liposomes [LIP(DMPC), LIP(F4-DMPC), and LIP(F8-DMPC)] bound to SBA24 (Figure 8). Figure 8A shows a Raman spectrum map of the C-H bond peak at 2800–3000 cm^−1^ for SBA24 with the bound liposomes. For LIP(DMPC) bound to SBA24, a strong C-H peak region was observed surrounding the particles [Figure 8A, LIP(DMPC)]. This indicated that the outer periphery of the particles was covered by liposomes. In the case of LIP(F4-DMPC)-conjugated SBA24, the intensity of the C-H peak surrounding the particles was slightly reduced [Figure 8A, LIP(F4-DMPC)]. In both cases, the background signals were low. In contrast, in the case of LIP(F8-DMPC)-bound SBA24, C-H peaks were observed over the entire particle. Furthermore, the C-H peak region was spread over the entire glass surface of the sample support. This suggested that LIP(F8-DMPC) was attached to not only the particles but also the glass surface, which was presumably hydrophobic, resulting in an overall high background signal. Figure 8B shows optical microscopy (OM) images of SBA24 with the bound liposomes shown in Figure 8A. Particles of almost the same shape and diameters of 3–5 µm were observed in each of the three types of samples. No differences due to the type of liposomes were observed under the optical microscope. Figure 8C shows the Raman spectrum of the SBA24 particles, as indicated by the arrow in the Raman spectrum map (Figure 8A). A peak originating from the C-H bonds of liposomes is apparent at 2800–3000 cm^−1^.

### 2.4. Qualitative Evaluation of Lipid Membranes Bound to Mesoporous SiO_2_ Microspheres via spICP-MS and CLRM

As described above, we successfully detected lipid membranes (liposomes and nanodiscs) bound to individual mesoporous SiO_2_ microspheres (SBA24) by using spICP-MS, targeting a specific element (P) contained in the lipid membranes (Figure 2, Figure 3, Figure 4, Figure 5, Figure 6 and Figure 7). The observed differences in binding frequency likely reflect not only size but also physicochemical properties. For liposomes, the degree of fluorination influences the hydrophobicity and membrane rigidity; F8-DMPC, with highly fluorinated chains, tends to form dense aggregates and may exhibit reduced flexibility, hindering pore penetration [5]. For nanodiscs, ND(2N2_DMPG) contains MSP2N2-His, a longer and more rigid scaffold protein than MSP1E3D1-His, and its negatively charged DMPG headgroups may enhance the electrostatic repulsion from the negatively charged silica surface at pH 7.4 [9]. Taken together, these findings suggest that the employed method is applicable to qualitative evaluation of the binding states of lipid membranes to individual porous particles. Previously, we showed that spICP-MS can be used to evaluate various characteristics of porous/nonporous particles, including the particle size and overall porosity [20]. Among these applications, we demonstrated that spICP-MS enables qualitative evaluation of the binding states of proteins bound to porous particles, including metal-binding proteins such as iron-binding lactoferrin and transferrin. The present findings extend the scope of this evaluation method to other biomolecules such as lipid membranes. Furthermore, the spICP-MS results were validated by visual and qualitative observations using CLRM (Figure 8). Thus, the method developed in this study offers a new platform for evaluating the effectiveness of particles as carriers of biomolecules (lipid membranes) and provides valuable insights for biomedical research and quality control in related industries.

These results indicate that spICP-MS with selective monitoring of the P contained in liposomes enables evaluation of the binding states of liposomes to porous particles. Notably, the ability to detect individual particles and assess their lipid content opens new possibilities for evaluating the distribution profiles across particle populations. This is particularly relevant for applications in which a uniform lipid coating is essential, such as drug-delivery carriers or diagnostic bead systems. By providing particle-level resolution, spICP-MS allows researchers and developers to compare the lipid-binding distributions between different formulations or processing conditions. This capability supports optimization of manufacturing parameters and may enhance quality-assurance practices, particularly in industries where batch-to-batch consistency is critical.

### 2.5. Potential Applications of Porous SiO_2_ Microspheres with Bound Liposomes as Biomembrane Mimetic Systems

The liposomes and nanodiscs investigated in this study are artificial systems that mimic biological membranes, making them useful tools for research and drug delivery. In particular, liposomes mimic cell membranes, as they form a lipid bilayer into which membrane proteins can be inserted. The advantage of using liposomes as membrane mimics is that they are fluid, allowing lateral mobility and self-healing [4]. These self-healing properties result from the free diffusion of lipids within the two leaflets of the lipid bilayer. Furthermore, liposomes are impermeable to ions and suppress non-specific binding of proteins. Liposomes have been used as embedding media for incorporating transmembrane proteins such as pore-forming proteins and ion channels. Moreover, the surfaces of liposomes can be modified by attaching biomolecules via covalent binding or biotin-avidin coupling [27,28,29,30]. The use of liposomes as biomimetic microspheres is problematic because of their instability. However, this is easily overcome by supporting lipid bilayers or monolayers on solid (nonporous) microspheres or porous SiO_2_ microspheres. This is accomplished by spontaneous adsorption, rupture, and fusion (as in this study) or by covalent modification of the sphere surface. In the case of porous SiO_2_ microspheres, the ordered pore network provides a large surface area and pore volume; in particular, the surface area was calculated to be approximately four orders of magnitude larger than that of solid microspheres of equivalent size [14,31]. This allows these microspheres to serve as carriers of biomolecules (such as lipid membranes and proteins), fluorescent dyes, and other chemical agents. In conclusion, the aforementioned properties of lipid bilayers within liposomes and their ability to incorporate membrane proteins make them appealing biomembrane mimetic systems that can be further stabilized by adsorption onto microspheres for use in various applications, such as biosensing.

## 3. Materials and Methods

### 3.1. Materials

Mesoporous SiO_2_ microspheres (SBA24 with a pore diameter of 23.5–23.6 nm) were synthesized in accordance with previously reported methods [32,33]. The dried SBA24 powder was stored at room temperature in a sealed desiccator until use. 10× Tris-buffered saline (TBS, pH 7.4; product code 317-90175) from Nippon Gene Corporation (Toyama, Japan) was diluted 10-fold with ultrapure water to prepare TBS. The solution was then used to suspend SBA24 and dilute the solutions of the liposomes and nanodiscs.

Liposomes are prepared using a variety of methods at temperatures above their gel-to-fluid transition temperature (*T*_m_) and vary in size from tens of nanometers to tens of micrometers, thus forming nano- or microspheres [4]. To prepare the liposomes used in this study, 1,2-dimyristoyl-sn-glycero-3-phosphocholine (DMPC) was purchased from Avanti Polar Lipids, Inc. (Alabaster, AL, USA), and the compounds 1,2-di(11,11,12,12,13,13,14,14,14-nonafluorotetradecanoyl)-sn-glycero-3-phosphocholine (F4-DMPC) and 1,2-di(7,7,8,8,9,9,10,10,11,11,12,12,13,13,14,14,14-heptadecafluorotetradecanoyl)-sn-glycero-3-phosphocholine (F8-DMPC) were synthesized according to previous reports [5,34]. Liposomes, including DMPC, F4-DMPC, and F8-DMPC, were prepared using a mini-extruder purchased from Avanti Polar Lipids, Inc., and stored at 3–6 °C in a refrigerator until use. Their properties are presented in Table 2. The diameters and polydispersity indices (PDIs) of the liposomes (Table 2) were determined using dynamic light scattering (Zetasizer Nano ZS; Malvern Instruments Ltd., Malvern, Worcs., UK). The determined diameter was the equivalent spherical hydrodynamic diameter, which included the electrical double layer around the liposome surface in solution and any other species linked to the liposome surface.

Nanodiscs, including MSP1E3D1-His_DMPC-biotinyl PE [ND(E3D1_DMPC)], MSP1E3D1_POPC-biotinyl PE [ND(E3D1_POPC)], and MSP2N2-His_DMPG [ND(2N2_DMPG)], were purchased from Cube Biotech (Monheim, Germany) and stored at −60 °C in a freezer until use. Their properties are presented in Table 3. The diameters of the nanodiscs (Table 3) were determined according to the MSPs used to create the discs, as reported by the manufacturer.

To determine the particle transport efficiency into the plasma in spICP-MS, suspensions of Pt nanoparticles (NPs) with nominal diameters of 30, 50, and 70 nm in aqueous 2 or 4 mM (for 70 nm Pt NPs only) citrate were purchased from nanoComposix (San Diego, CA, USA) and used as particle standards. The mean diameters and standard deviations of the NPs, as determined via transmission electron microscopy (JEOL 1010; JEOL, Tokyo, Japan), were (31 ± 3), (46 ± 5), and (70 ± 4) nm, respectively. To create a particle standard calibration curve (four points including zero) using spICP-MS, particle standard solutions with a particle number concentration of approximately 1 × 10^5^ particles/mL were prepared from the original particle standards.

To create a solution standard calibration curve using spICP-MS, ion standard solutions of P with different concentrations of 0–100 μg/L (three points) and of Pt with different concentrations of 0–10 μg/L (four points) were prepared from standard solutions of 1000 mg/L phosphate ion (PO_4_^3−^) and 1000 mg/L Pt (Kanto Chemical Corporation, Tokyo, Japan), respectively.

### 3.2. Sample Preparation

Approximately 1 mg of dried SBA24 powder was weighed in a tube. One milliliter of aqueous TBS buffer (pH 7.4) was added to the tube and rigorously vortexed twice for 3 s each time. The resultant suspension was gently rotated at 20–25 °C for 5 min for SiO_2_ equilibration. The SBA24 suspension was centrifuged at 19,000× *g* for 1 min at 20 °C, the supernatant was carefully removed, and the remaining pellet was used as TBS-equilibrated SBA24 for further experiments.

Three different types of liposomes [LIP(DMPC), LIP(F4-DMPC), and LIP(F8-DMPC)] and nanodiscs [ND(E3D1_DMPC), ND(E3D1_POPC), and ND(2N2_DMPG)] were used as representative lipid membranes to bind SBA24. Approximately 10 µL of LIP/ND was placed in each 15 mL tube. TBS (4990 µL) was added to each tube for dilution to 5 mL and vortexed twice for 3 s each. The resulting solution was used as the LIP/ND solution to prepare LIP/ND-bound SBA24.

The adsorption of the liposomes onto the pores of SBA24 was performed by combining 1 mL of the LIP solution containing LIP(DMPC) (73.8 mM as lipid), LIP(F4-DMPC) (50.7 mM as lipid), and LIP(F8-DMPC) (39.5 mM as lipid), respectively, with 1 mg of TBS-equilibrated SBA24. The adsorption of the nanodiscs onto the pores of SBA24 was performed by combining 1 mL of the ND solution containing ND(E3D1_DMPC) (1 µM), ND(E3D1_POPC) (1 µM), and ND(2N2_DMPG) (0.9 µM), respectively, with 1 mg of TBS-equilibrated SBA24. The LIP/ND-SBA24 mixtures were gently agitated using a rotator for 1 h at 20–25 °C and centrifuged at 19,000× *g* for 1 min at 20 °C. After centrifugation, the LIP/ND-bound SBA24 was rinsed twice with 1 mL of TBS for subsequent qualitative evaluation via spICP-MS and CLRM.

### 3.3. Qualitative Evaluation of Liposomes and Nanodiscs Bound to Mesoporous SiO_2_ Microspheres via spICP-MS

A quadrupole ICP-MS instrument (Agilent 7700x ICP-MS; Agilent Technologies, Santa Clara, CA, USA) equipped with an ICP torch having an injector tube of diameter 1.5 mm, a concentric MicroMist nebulizer with a natural sample uptake rate of 400 µL/min, a standard sample tube with a 0.5 mm inner diameter, and a Scott double-pass spray chamber cooled at 2 °C was used for spICP-MS, in combination with an externally assembled high-speed pulse signal-processing system [35]. The ICP-MS instrument was tuned daily using a tuning solution containing 1 ng/mL each of Li, Co, Y, Ce, and Tl in 2% nitric acid (HNO_3_) to achieve optimum signal intensity and stability. The typical operating conditions of the ICP-MS instrument are presented in Table 1. Measurements were conducted in the He mode at a dwell time of 100 µs. All samples were measured three times for a 60 s period each to ensure the detection of a sufficient number of particles, which enabled the attainment of statistically reliable results. Between samples, the instrument was cleaned with 2% HNO_3_ for 90 s.

The measurements of the elemental (P) masses of the lipid membranes per particle using spICP-MS were based on a conventional calibration approach using an ion standard solution (i.e., the ion standard solution approach) [36,37]. This approach employs a mass flux calibration curve from phosphate ion (PO_4_^3−^) standard solutions and determines the elemental (P) masses of the lipid membranes per particle. Briefly, a calibration curve was constructed by relating the P concentrations of the phosphate ion standard solutions to the ^31^P signal intensity. The P concentration of the standard solution was then converted to the mass flux using the following expression:

*W* = *C*_STD_ × *Q*_neb_ × *t*_dwell_ × *η* (1)

Here, *W* is the delivered mass per unit dwell time (ng), *C*_STD_ is the mass concentration (ng/g), *Q*_neb_ is the sample flow rate (g/s), *t*_dwell_ is the dwell time (s), and *η* is the transport efficiency (%). The mass concentration, sample flow rate, dwell time, and transport efficiency were determined experimentally. The actual sample flow rate based on the nebulizer pump speed set at 0.10 rps was 0.332 g/min. The transport efficiency calculated based on the particle size method [36] was 8.7%, as described in detail below. The integrated ^31^P signal intensity (i.e., peak area) of each particle event was then substituted into the resulting mass-flux calibration curve (three points including zero) with a slope of 2.8 × 10^9^ counts/μg, an intercept of 15.3 counts, and a correlation coefficient (*R*^2^) of 0.9974. Thereby, the elemental (P) masses of the lipid membranes bound to the corresponding particles were obtained.

The transport efficiency is defined as the ratio of the amount of analyte entering the ICP system to the amount of aspirated analyte. In this study, the particle size method explored by Pace et al. [36] was applied to determine the transport efficiency. The transport efficiency calculated by the method using both the solution standard calibration curve (with a slope of 5.1 × 10^9^ counts/μg) and the particle standard calibration curve (with a slope of 5.9 × 10^10^ counts/μg) for ^195^Pt was calculated to be 8.7%. This value was validated by comparing it with the transport efficiency value (8.7% ± 0.2%, mean ± standard deviation, *n* = 3) calculated based on the particle frequency method [36] using Pt NPs with a nominal size of 50 nm.

### 3.4. Qualitative Evaluation of Liposomes Bound to Mesoporous SiO_2_ Microspheres via CLRM

The suspension (10 µL) of SBA24 with the bound liposomes [LIP(DMPC), LIP(F4-DMPC), and LIP(F8-DMPC)] was dropped onto a slide glass, and the top was sealed with a cover glass using a 5-µm-thick double-sided tape as a spacer. The samples were examined via CLRM using a 532 nm Nd:YAG laser (alpha300R; WITec, Ulm, Germany). The spectra were acquired using a Peltier-cooled charge-coupled device detector (DV401-BV, Andor Technology Ltd., Belfast, UK) at 600 g/mm (UHTS 600; WITec). The Raman data were analyzed using the WITec suite (version 5.0; Lab Co., Northampton, MA, USA) and MATLAB R2024a (MathWorks Inc., Natick, MA, USA). For observation of SBA24 particles, a 20 µm × 20 µm area at the cell center was scanned at 100 pixels × 100 pixels using a 50× objective lens at a laser intensity of 15 mW at 50 ms for each pixel. This device included a 100-µm-diameter optical fiber and the ~20-µm-diameter core functioned as a pinhole.

## 4. Conclusions

The results of this study indicate that spICP-MS can be used to evaluate the binding states of lipid membranes to mesoporous SiO_2_ microspheres. In a previous study [21], we investigated the applicability of spICP-MS for the measurement of SiO_2_ microspheres and the detection of iron-containing proteins (i.e., lactoferrin and transferrin) bound to mesoporous SiO_2_ microspheres. We conclude that spICP-MS is applicable to the particle-size measurement of nonporous/mesoporous SiO_2_ microspheres and has considerable potential for element-specific detection and qualification of proteins bound to mesoporous SiO_2_ microspheres in various applications. This method offers a new platform for not only the qualitative evaluation of lipid membranes and proteins bound to particles but also the evaluation of the effectiveness of particles as carriers for biomolecules. Thus, it can provide valuable insights for biomedical research and quality control in related industries.

In particular, the ability to assess lipid-membrane binding at the single-particle level allows the detection of distribution patterns and particle-to-particle variability in the lipid content, which cannot be detected through conventional bulk analysis. This capability is particularly relevant in contexts where the uniformity and reproducibility of surface functionalization are critical, such as the development of biofunctionalized particles for therapeutic or diagnostic use. The sensitivity of the method and element-specific detection also allow comparative evaluation across different lipid-membrane types and particle formulations, supporting the optimization of experimental conditions and formulation strategies. These features make spICP-MS a promising analytical tool for both exploratory research and applied studies involving lipid membrane–particle systems.

In future studies, we aim to explore quantitative extensions of this approach and expand its application to other biomolecular systems, including those relevant to sustained-release formulations, vaccine carriers, and biosensing technologies. Such efforts will further establish spICP-MS as a versatile platform for characterizing complex biointerfaces at the particle level.

## Figures and Tables

**Table 1:** Typical operating conditions of the ICP-MS instrument.

Parameter	Setting
Plasma and sampling conditions	
Radiofrequency power	1550 W
Plasma gas flow rate	15 L/min
Auxiliary gas flow rate	0.90 L/min
Carrier (nebulizer) gas flow rate	0.90 L/min
Nebulizer pump	0.10 rps
Sampling position	10.0 mm
Cell gas (He) flow rate	0.0 mL/min for ^13^C and ^31^P 3.0 mL/min for ^28^Si 5.0 mL/min for ^12^C
Data acquisition	
Scanning mode	Peak hopping
Number of data points per peak	1
Monitored isotopes	^12^C, ^13^C, ^28^Si, ^31^P

**Table 2:** Properties of the liposomes used in this study, including their diameters, PDIs, lipid concentrations, molecular weights, and phase transition temperatures (*T*_m_).

	Diameter (nm)	PDI	Lipid Concentration (mg/0.5 mL)	Lipid Molecular Weight (g/mol)	Lipid *T*_m_ (°C)
LIP(DMPC)	138.2	0.056	25.0	677.94	25.0
LIP(F4-DMPC)	136.3	0.181	25.4	1001.77	5.4
LIP(F8-DMPC)	337.4	0.156	25.5	1289.62	64.4

**Table 3:** Properties of the nanodiscs used in this study, including their diameters, MSPs, phospholipids, and concentrations.

	Diameter (nm)	MSP	Phospholipids	Concentration (µM)
ND(E3D1_DMPC)	~12–14	MSP1E3D1-His	1,2-dimyristoyl-sn-glycero-3-phosphocholine (DMPC) + 10% 1,2-dioleoyl-sn-glycero-3-phosphoethanolamine-N-(biotinyl) (Biotinyl-PE 16:0) headgroup-modified lipid	500
ND(E3D1_POPC)	~12–14	MSP1E3D1-His	1-palmitoyl-2-oleoyl-sn-glycero-3-phosphocholine (POPC) + 10% 1,2-dioleoyl-sn-glycero-3-phosphoethanolamine-N-(biotinyl) (Biotinyl-PE 18:1) headgroup-modified lipid	500
ND(2N2_DMPG)	~17	MSP2N2-His	1,2-dimyristoyl-sn-glycero-3-phosphoglycerol (DMPG)	500

## Data Availability

The original contributions presented in this study are included in the article. Further inquiries can be directed to the corresponding author.
